# A crash course in sequencing for a microbiologist

**DOI:** 10.1007/s13353-019-00482-2

**Published:** 2019-01-25

**Authors:** Aleksandra Kozińska, Paulina Seweryn, Izabela Sitkiewicz

**Affiliations:** 1 Department of Drug Biotechnology and Bioinformatics, National Medicines Institute, Chelmska 30/34, 00-725 Warszawa, Poland; 2 Department of Microbiology and Antibiotics, National Medicines Institute, Chelmska 30/34, 00-725 Warszawa, Poland

**Keywords:** Sanger sequencing, Next-generation sequencing, Structural genomics, Transcriptomics, Phylogenetic analysis, Microbiome

## Abstract

For the last 40 years, “Sanger sequencing” allowed to unveil crucial secrets of life. However, this method of sequencing has been time-consuming, laborious and remains expensive even today. Human Genome Project was a huge impulse to improve sequencing technologies, and unprecedented financial and human effort prompted the development of cheaper high-throughput technologies and strategies called next-generation sequencing (NGS) or whole genome sequencing (WGS). This review will discuss applications of high-throughput methods to study bacteria in a much broader context than simply their genomes. The major goal of next-generation sequencing for a microbiologist is not really resolving another circular genomic sequence. NGS started its infancy from basic structural and functional genomics, to mature into the molecular taxonomy, phylogenetic and advanced comparative genomics. Today, the use of NGS expended capabilities of diagnostic microbiology and epidemiology. The use of RNA sequencing techniques allows studying in detail the complex regulatory processes in the bacterial cells. Finally, NGS is a key technique to study the organization of the bacterial life—from complex communities to single cells. The major challenge in understanding genomic and transcriptomic data lies today in combining it with other sources of global data such as proteome and metabolome, which hopefully will lead to the reconstruction of regulatory networks within bacterial cells that allow communicating with the environment (signalome and interactome) and virtual cell reconstruction.

## Introduction

A little over 40 years ago, Fred Sanger published his paper describing dideoxy chain terminator sequencing (Sanger et al. 1977). This first genome sequencing of bacteriophage φX174 was performed using long sequencing gels and radioactively labeled nucleotides. In addition, gels needed exposure to photographic film and manual sequence reading. Only later development of fluorescence-based methods and automated instrumentation allowed to increase speed and the scale of dideoxy chain terminator sequencing (Smith et al. 1986).

For over 30 years, “Sanger sequencing” allowed to unveil crucial secrets of life. The technology was used to sequence in 1981 first part of the human genome—mitochondrial DNA (Anderson et al. 1981)—and years later first complete bacterial genome of *Haemophilus influenzae* (Fleischmann et al. 1995) A decade between the mid-1990s and mid-2000s gave us genomic sequences of other bacterial species, and we saw the rise of comparative genomics and metagenomics (for a review, see Loman and Pallen (2015)). Those early years were crucial not only for gaining basic knowledge about bacterial genomes, but also to develop sequencing strategies and lay the foundation for future microbial genomics and transcriptomics. Sequencing of bacterial genomes also prompted the development of bioinformatics and software required to handle a large amount of data.

Although dideoxy chain terminator sequencing was absolutely critical in the development of modern microbiology, from molecular to environmental sciences, the method itself has been relatively troublesome. Sequencing was time-consuming and laborious, especially in the years before the introduction of cycle sequencing (Murray 1989), and remains expensive even today. Human Genome Project was a huge impulse to improve sequencing technologies, and unprecedented financial and human effort prompted the development of cheaper, less time-consuming, and high-throughput technologies and strategies.

**Fig. 1:**
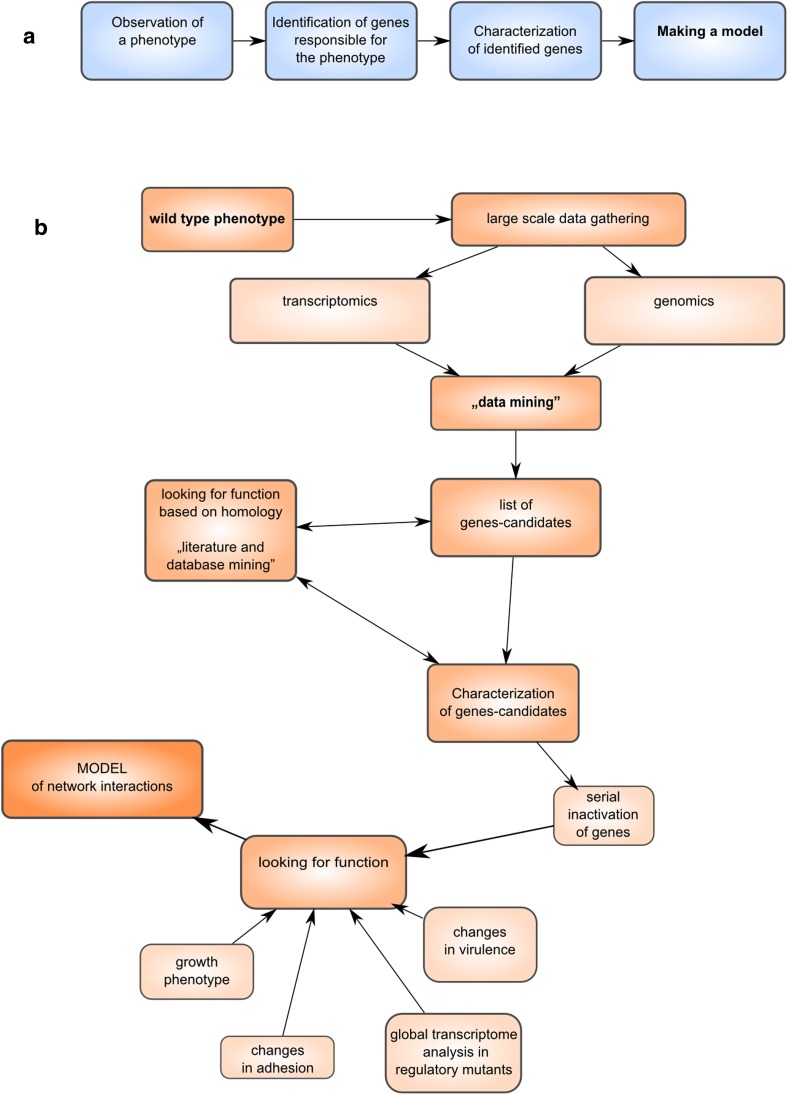
Differences of experimental approach between classical (**a**) and experiments involving high-throughput analyses such as NGS (**b**)

This review will discuss some applications of high-throughput methods to study bacteria in a much broader context than simply their genomes. It could be even stated that nowadays the major goal of next-generation sequencing is not really resolving another circular genomic sequence; NGS is simply another tool that can be used to characterize bacteria in a very broad context. We can use NGS in detailed investigations and large-scale epidemiological studies. However, the major influence of affordable high-throughput methods on modern microbiology is a change of the way we think about the experimental approach (Fig. 1). The way we test hypotheses, plan experiments, and make conclusions, have changed. Instead of simple reasoning that leads from single observations of biological phenomena to generating a model of activity (Fig. 1a), multiple high-throughput methods, including genomic studies, combined with data mining, allow drawing a broad picture (Fig. 1b).

## Technologies

Because a number of excellent comparisons of next-generation sequencing technologies are available (Buermans and den Dunnen 2014; Goodwin et al. 2016; Heather and Chain 2016; Levy and Myers 2016; Metzker 2010), this review will rather concentrate on applications of those sequencing methods in microbiology, than the description of technologies and economic aspects of such experiments. In general, over the last decade, we could observe the constant development of methods and changes in sequencing chemistry that produced simplified, more efficient instruments that allow to cheaply generate a tremendous amount of data. Costs of sequencing dropped dramatically with the development of new sequencing technologies. Within the last 10 years, a cost to determine a sequence of a human exome was lowered over 15,000 times, from about 15 million USD to below 1000 dollars (https://www.genome.gov/27565109/the-cost-of-sequencing-a-human-genome/). It makes the technology usable in multiple applications that require high-volume output. As Michael L. Metzker wrote in his review of sequencing technologies “… the potential of NGS is akin to the early days of PCR, with one’s imagination being the primary limitation to its use…” (Metzker 2010). And we would like it to be a take-home message for all the readers of this review. It is not the technology we should focus on, but we should rather expand our research and gather more complex data that can be used to answer multiple questions at the same time. It is only our imagination that can lead us to scientific discoveries.

First attempts to modify and make sequencing technologies more flexible started in the late 1980s (Hultman et al. 1989) and continued through the whole decade of 1990s (Nyren et al. 1993). In the early 1990s, multiple scientists from large academic hubs such as Cambridge or Harvard University started developing new sequencing methods based on different principles than Sanger sequencing. The early technologies included polony sequencing (Shendure et al. 2005), pyrosequencing (Margulies et al. 2005), and early single molecule sequencing (Helicos BioSciences). This whole scientific movement promoted the development of new technologies and, as a consequence, made the new technologies available on the market. In recent years, more sequencing technologies such as single molecule real-time sequencing or nanopore sequencing have been developed (Table 1) and are currently on the market or pre-market stage. There are new platforms from “old” players like Qiagen (GeneReader), Roche (Roche Genia), or Illumina (NovaSeq), but also completely new technologies are being developed. New sequencing technologies utilize multiple approaches like quantum (http://quantumbiosystems.com) or microdroplet/microfluidic sequencing (http://www.base4.co.uk).

**Table 1:** Comparison of sequencing technologies available currently on the market, based on Liu et al. (2012), Quail et al. (2012), Pevsner (2015), Levy and Myers (2016)) and information from system manufacturers (https://www.thermofisher.com, https://nanoporetech.com/, http://sequencing.roche.com, https://www.illumina.com/, https://www.pacb.com/)

Method	Instrument manufacturer	Read length	Accuracy (single read not consensus)	Reads per run	Time per run (not including library preparation)	Cost per 1 million bases (in US$)	Advantages	Disadvantages
Chain termination (Sanger sequencing)	ThermoFisher	400 to 900 bp	99.9%	N/A	20 min to 3 h	$2400	Useful for many smaller applications	Very expensive
Ion semiconductor (ion torrent sequencing)	ThermoFisher	Up to 600 bp	98%	Up to 80 million	2 h	$1	Less expensive NGS equipment. Fast	Homopolymer errors
Nanopore sequencing	Oxford Nanopore	Depends on the library preparation, not the device	~ 92–97%	Dependent on read length selected by user	Data streamed in real time. No fixed running time	$500–999 per flow cell	Long individual reads. Portable (palm-sized)	Lower throughput than other machines, low single read accuracy
Pyrosequencing	Roche	700 bp	99.9%	1 Million	24 h	$10	Long reads. Fast	Runs are expensive. Homopolymer errors
Sequencing by synthesis	Illumina	50–500 bp depending on the instrument	99.9%	1 Million–3 billion depending on the instrument	1 to 11 days, depending upon sequencer and specified read length	$0.05 to $0.15	High output. Low costs of sequencing	Expensive equipment
Sequencing by ligation (SOLiD sequencing)	ThermoFisher	50 + 35 or 50 + 50 bp	99.9%	1.2 to 1.4 Billion	1 to 2 weeks	$0.13	Low cost per base	Slower than other methods. Problems with palindromic sequences
Single-molecule real-time sequencing	Pacific Biosciences	On average 14,000 bp maximum read length > 40,000 bases	87% Single-read accuracy	50,000 per SMRT cell, or 500–1000 megabases	30 min to 4 h	$0.13–$0.60	Fast. Long reads	Moderate throughput. Equipment is very expensive

## High-throughput methods for a microbiologist

### The infancy and childhood—structural and functional genomics

Early microbial sequencing projects tried to simply answer the questions about the nucleotide sequence and attempted to elucidate gene function, predominantly based on homology to known genes. Hence, the first attempts can be rather simply described as structural and early functional genomic projects. The biggest problem, however, for microbial and structural genomics since those early attempts is genome annotation. Often, open reading frames were misidentified, not much has been also known about basic transcription and translation in non-model microorganisms. Only recent use of NGS in transcriptomic approaches (see below) allows to answer those early questions about the basic biology of microorganisms.

Annotation and assigning function based only on homology is often misleading as genes of the similar sequence can have completely different functions in various microorganisms. It is especially true for regulatory genes; their specificity and biological function can be rarely predicted based on the sequence alone. Therefore, proper annotation of at least model organisms should include functional analysis. So far, the best microbial annotation is available for *Escherichia coli* K12 MG1655 in EcoCyc (https://ecocyc.org/) database. It includes over 85% of genes with experimentally confirmed function (Karp et al. 2007; Keseler et al. 2017). EcoCyc also contains a reconstruction of metabolic networks, cellular trafficking, and regulatory processes. Decades of research on *E. coli* generated combined knowledge that can be further utilized from modeling and network prediction using bioinformatic tools (Feist et al. 2009). Because multiple bacterial species are not as well studied as *E. coli*, they usually contain a large percentage of genes of unknown function encoded by their genome. Their annotation is predominantly based on gene ontology, but also advanced algorithms to reconstruct metabolic pathways and assign gene function based on functional reconstruction are used. Examples of such tools that allow predicting gene function with high probability are RAST (Aziz et al. 2008) or Kyoto Encyclopedia of Genes and Genomes (KEGG) (Kanehisa et al. 2016). In the opinion of many microbiologists that analyze genomic data, all the early sequencing projects require extensive re-working, corrections, and additional functional information.

### Teenage years and early adulthood—taxonomic and phylogenetic studies beyond comparative genomics

**Fig. 2:**
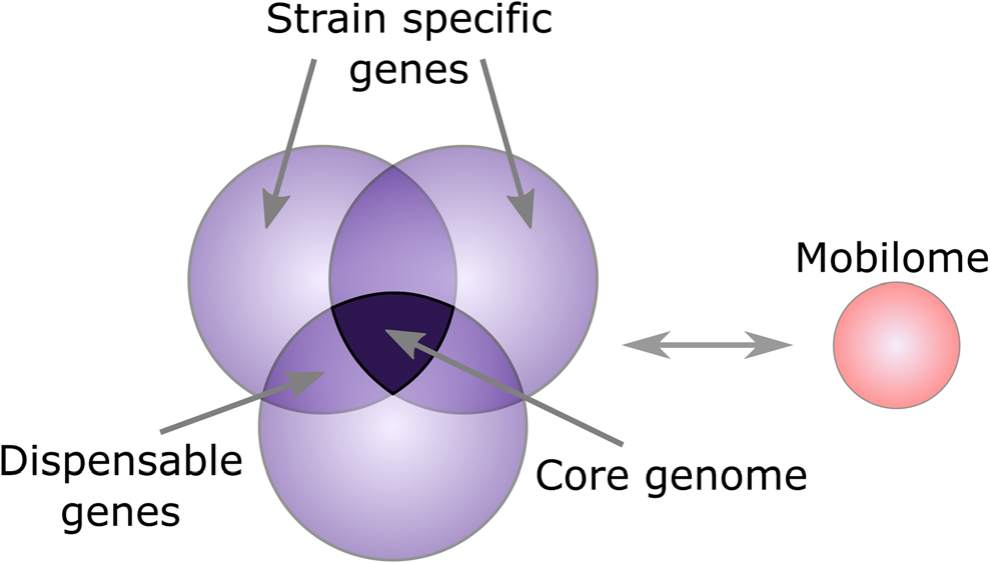
A schematic structure of a pan-genome that includes genes shared by all strains/isolates, genes shared by all genomes (core genome), and strain-specific genes that are present only in individual

Those early sequencing projects quickly expanded into comparative genomic field as the number of sequenced genomes grew. Initially, as a consequence of a lack of a large number of fully sequenced genomes, only comparisons between species were performed (for a review, see Loman and Pallen (2015)). Later with expanding the number of genomic sequences, first intra-species comparisons were performed, such as comparison of two *Helicobacter pylori* strains (Alm et al. 1999). Further accumulation of sequence data reflecting the bacterial diversity within the species allowed to define genomic elements that shape the bacterial community and changed the perspective on the definition of bacterial species (Gupta 2016; Caro-Quintero and Konstantinidis 2012; Gao and Gupta 2012) (Fig. 2).

The decreasing cost of sequencing inevitably changed microbial taxonomy and allowed to sequence and compare thousands of genomic sequences available for single bacterial species (Nasser et al. 2014; Long et al. 2017).

Since the genome itself is no longer a sole goal and costs of NGS are much lower, multiple techniques requiring sequencing are increasingly switching to NGS. A big field that utilizes sequencing techniques is microbial diagnostics. For years, typing techniques to determine unique strain characteristics included restriction fragment analysis (RFLP-PFGE), analysis of polymorphic sites such as MLVA, SNP detection by sequencing, high-resolution melting curves, detection of virulence, and antibiotic resistance genes (for a review of the typing methods, see Li et al. (2009)). Even a few years ago, NGS was too expensive to replace those methods for routine diagnostics in the microbiology laboratory. Today, many larger laboratories switch to NGS and other molecular diagnostic methods to increase the speed of proper diagnosis (Opota et al. 2015). Many companies produce user-friendly software to speed up typing and increase the sensitivity of detection dedicated to microbiology research (http://www.ridom.de/seqsphere/). It allows to detect all polymorphisms, structural changes, and mobile genetic elements and reconstruct outputs of traditional typing methods such as RFLP-PFGE solely based on the genomic sequence. Such a detailed analysis can be used for advanced correlation studies such as GWAS (genome-wide association studies) to detect a connection between polymorphisms and disease manifestation or severity (Chen and Shapiro 2015). Diagnostic microbiology also utilizes amplicon-based profiling that allows to sequence selected amplicons such as regions encoding 16S rRNA that are used for species identification. Kits and protocols for 16S sequencing are commercially available and easy to apply. It also allows to perform virtual equivalents of other PCR typing methods such as MLVA-MLVF, detect certain virulence traits, or analyze highly polymorphic loci using user-designed amplicons.

Diagnostic laboratories routinely assay for antibiotic resistance of bacterial strains isolated from patients. The major disadvantage of standard procedure to determine minimal inhibitory concentration is the length of the procedure. It usually requires pure culture that can take over 24 h and growth test that takes from 16 to 24 h. DNA-based methods to detect determinants of antibiotic resistance are much faster, but they do not give much information about MIC value. Recently, NGS sequencing was employed for in silico prediction of MICs for selected antibiotics based on the genomic sequence (Nguyen et al. 2018). MICs can be predicted based on cell properties such as the presence of a certain combination of resistance determinants and membrane transporters. The ability to predict MICs based on genomic data completely changes the outlook on the modern diagnostics microbiology.

Another important application of NGS is tracing epidemics in real time. It can be used as a clinical tool to trace transfers of bacteria in the hospital settings as part of epidemiological investigations (Long et al. 2014). To date, confirmation that epidemic strains isolated from infected subjects are clones of the same strain required laborious investigation. For example, Pulse Net (https://www.cdc.gov/pulsenet/index.html)—a network dedicated to the detection of foodborne infections causing outbreaks, for years—have used RFLP-PFGE to establish a connection between strains. Recently, PulseNet introduced sequencing methods to establish clonal outbreaks. Improvement of portable instruments such as MinION enables rapid in situ diagnostics and creates a stream of genomic data that can reveal critical epidemiological aspects of an outbreak or epidemics dynamics. By coupling sequencing to an enhanced surveillance and response platform, a more anticipatory approach to outbreak prevention and control can be applied (Gardy and Loman 2017).

Sequencing data are used by global networks such as the global microbial identifier (GMI) (http://www.globalmicrobialidentifier.org/). GMI is focused on the improvement of a global system of DNA genome databases for microbial and infectious disease identification and diagnostics. One of the goals of the network is early detection of outbreaks and tracking outbreaks in real time. Institutions involved in this project include Food and Drug Administration (FDA), World Health Organization (WHO), Food and Agriculture Organization (FAO), World Organization of Animal Health (OIE), and National Center for Biological Information (NCBI), public health agencies from multiple European countries, Thailand, Singapore, Japan, China.

Next level of comparative genomics utilizes large datasets generated using NGS allow to perform sophisticated comparative evolutionary analyses of multiple layered networks such as epistasis analysis detecting interactions and co-evolution of virulence or resistance traits in bacteria (Skwark et al. 2017). However, NGS technologies are increasingly more often utilized for various analyses of gene expression.

### Maturity—transcriptomics and microbiome analysis

Transcriptomics started in the early 1980s from quantitative and qualitative studies on single transcripts using northern analysis and later making cDNA libraries and studying expressed sequence tags (EST). From the early on, researchers interested in studying the transcriptome focused rather on eukaryotic than bacterial genes. A massive effort was put into sequencing cDNA libraries and later development of techniques such as SAGE (serial analysis of gene expression) (Velculescu et al. 1995). The next big step in the transcriptome analysis was the development and improvement of quantitative PCR (see references in Kralik and Ricchi (2017)) and later microarrays (Davenport et al. 2017). Today, most of these techniques are currently being replaced by RNA sequencing technologies. Quantitative PCR, and microarrays are rather used for the detection of genes or mutations, rather than transcriptomic studies. Multiple transcriptomic studies in bacteria changed the outlook on the gene regulation in bacteria (see below) and prompted, in turn, massive changes in our understanding of basic biological processes in bacteria.

The improvement of RNA sequencing technologies expands the capabilities of the method. Today, sequencing requires small amount of initial material and allows to investigate the coordination of gene expression in vivo, during the infection (Beres et al. 2016), or easily correlate in vivo and in vitro investigations to study the role of particular regulators in virulence (Eraso et al. 2016; Calfee et al. 2017). Extensive genome sequencing followed by transcriptomic studies allowed the identification of massive antisense transcription in bacteria, as well as the presence of multiple small regulatory RNAs that play important role in the regulation of virulence (Georg and Hess 2018; Figueroa-Bossi and Bossi 2018).

**Fig. 3:**
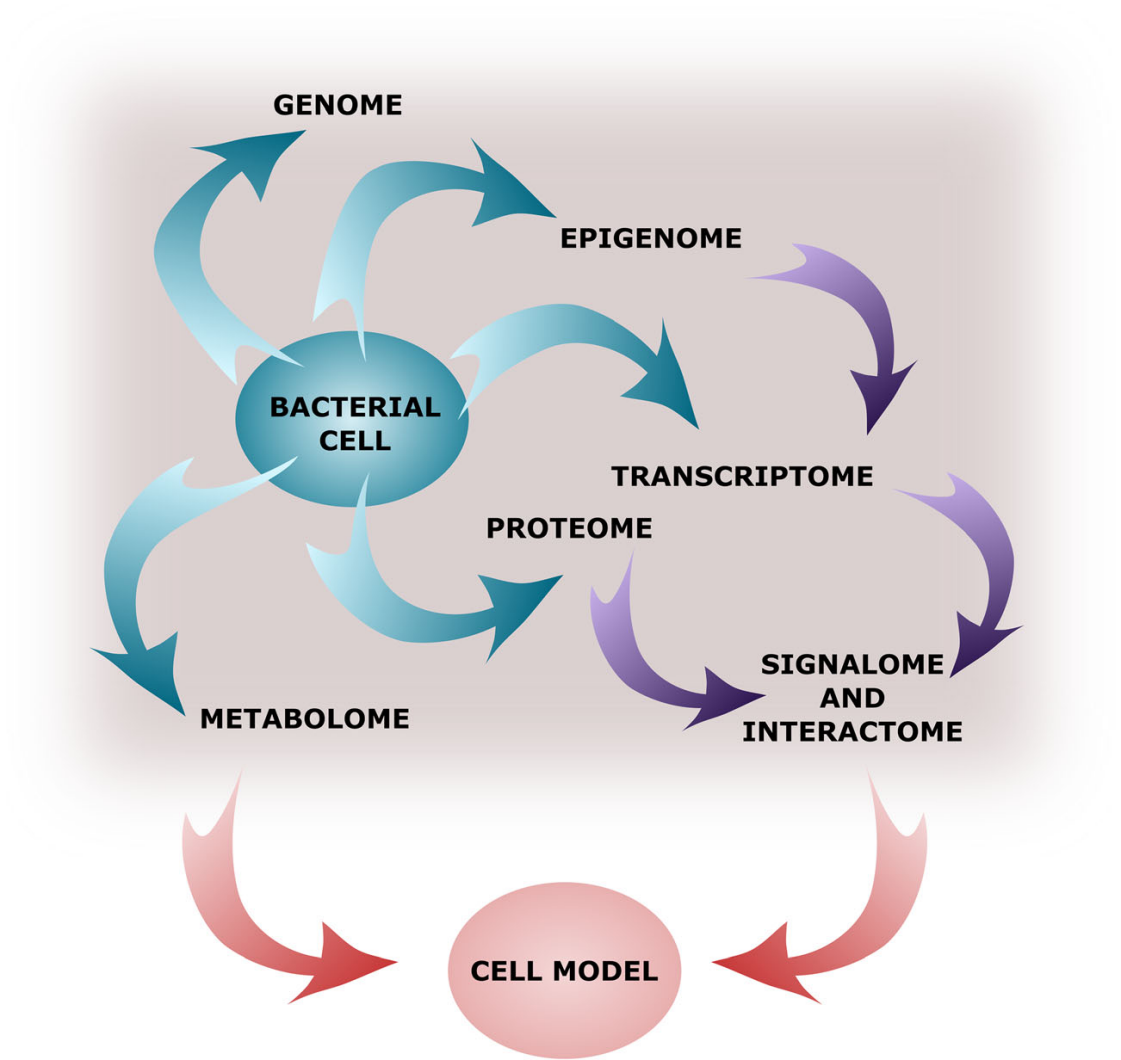
A virtual cell reconstruction that will be possible in the future based on a combination of multiple levels of the “-omics” data

RNA sequencing allows to expand studies on basic bacterial biology such as transcription, translation, and the interaction of basic molecules in the living cells (Fig. 3) and link multiple levels of information such as genomic and transcriptomic data.

Another direction in microbiology possibly thanks to NGS is a complex analysis of microbial communities. It started as simply as attempts to quantitatively assess composition and proportions of species within various microbiomes. The preferred method for such analysis has been 16S amplicon sequencing as a relatively inexpensive way to establish community composition. Similar studies utilizing sequencing of certain regions such as ITS2 and/or 18S RNA were also conducted to establish human mycobiome (Nash et al. 2017).

In recent few years, with lower sequencing costs, the scale of microbiome sequencing could be increased to the whole metagenome instead of 16S amplicon sequencing. It allows for example to study the metabolic properties of the bacterial community or co-evolution of the host and bacteria (Davenport et al. 2017; Peisl et al. 2017). This approach allows to characterize unique bacterial communities and bacteria that are unable to grow in laboratory conditions, which often leads to discoveries of new active molecules such as antibiotics (Hover et al. 2018; Charlop-Powers et al. 2016; Ling et al. 2015). The availability of the whole genome sequences of the microbiome prompted also the rapid development of microbiome metatranscriptomics, especially for the microbiota and gut interactions (Lavelle and Sokol 2018; Bashiardes et al. 2016).

The opposite trend in sequencing is the increase of sequencing sensitivity that allows to sequence material isolated from single cells (Gawad et al. 2016). Single cell genomics allows understanding unique components of the complex microbial ecosystems. In addition, single-cell microorganism sequencing has enabled the genome assembly of new phyla and is beginning to provide new biological insights into the microbial world we had no idea exists. The ability to sequence DNA from single cells also allows studying transcriptome on the single-cell level.

## Summary

Within the last 2–3 years, we have witnessed a rapid increase in quantity and quality in genomic and transcriptomic research that expands into other “-omics” fields. Technological changes allowed the expansion of our knowledge and changed views on bacterial genetics and biology. Our views, especially on the mechanisms of gene expression in bacteria, changed. Even operon concept is no longer considered “simple” as a vast array of alternative regulatory mechanisms has been detected. Multiple “eukaryotic” regulatory mechanisms such as alternate transcripts, non-coding RNAs, overlapping UTRs, leaderless mRNA, riboswitches, antisense RNA, regulation by genome structure, or epigenetic modifications are being detected in bacteria (for a review, see Güell et al. (2011) and Sánchez-Romero et al. (2015). The new discoveries and downstream studies are in the vast majority possibly thanks to the development of the new sequencing techniques.

The major challenge in understanding genomic and transcriptomic data lies today in combining it with other sources of global data such as proteome and metabolome, which hopefully will lead to the reconstruction of regulatory networks within bacterial cells that allow communicating with the environment (signalome and interactome) and virtual cell reconstruction (Fig. 3).

**Fig. 4:**
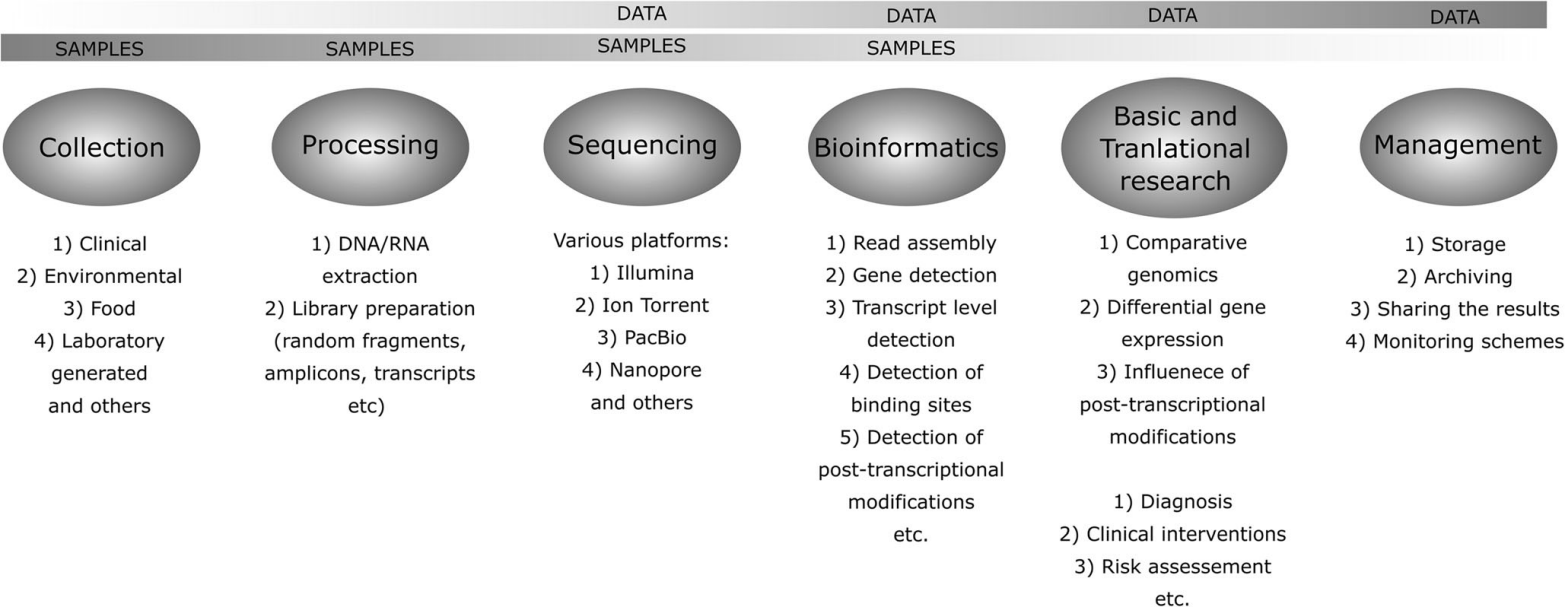
Overview of the different steps involved in the use of NGS technologies for data gathering and utilization. After Angers-Loustau et al. (2018), modified

The NGS techniques involve multiple layers of sample collection, preparation, and analysis (Fig. 4). The use of the sequencing data goes beyond hardcore basic science, but is also a base for the translational research and can be used as a diagnostic tool, in treatment risk assessment or clinical interventions.
